# Design of Proposed Software System for Prediction of Iliosacral Screw Placement for Iliosacral Joint Injuries Based on X-ray and CT Images

**DOI:** 10.3390/jcm12062138

**Published:** 2023-03-09

**Authors:** Vojtech Benda, Jan Kubicek, Roman Madeja, David Oczka, Martin Cerny, Kamila Dostalova

**Affiliations:** 1 Department of Cybernetics and Biomedical Engineering, VŠB—Technical University of Ostrava, 17. listopadu 2172/15, Poruba, 708 00 Ostrava, Czech Republic; 2 Trauma Center, University Hospital Ostrava, 17. listopadu 1790, Poruba, 708 52 Ostrava, Czech Republic

**Keywords:** iliosacral screw, multimodal image registration, multiregional image segmentation, DDR projection, software materialize mimics

## Abstract

One of the crucial tasks for the planning of surgery of the iliosacral joint is placing an iliosacral screw with the goal of fixing broken parts of the pelvis. Tracking of proper screw trajectory is usually done in the preoperative phase by the acquisition of X-ray images under different angles, which guide the surgeons to perform surgery. This approach is standardly complicated due to the investigation of 2D X-ray images not showing spatial perspective. Therefore, in this pilot study, we propose complex software tools which are aimed at making a simulation model of reconstructed CT (DDR) images with a virtual iliosacral screw to guide the surgery process. This pilot study presents the testing for two clinical cases to reveal the initial performance and usability of this software in clinical conditions. This model is consequently used for a multiregional registration with reference intraoperative X-ray images to select the slide from the 3D dataset which best fits with reference X-ray. The proposed software solution utilizes input CT slices of the pelvis area to create a segmentation model of individual bone components. Consequently, a model of an iliosacral screw is inserted into this model. In the next step, we propose the software CT2DDR which makes DDR projections with the iliosacral screw. In the last step, we propose a multimodal registration procedure, which performs registration of a selected number of slices with reference X-ray, and based on the Structural Similarity Index (SSIM) and index of correlation, the procedure finds the best match of DDR with X-ray images. In this pilot study, we also provide a comparative analysis of the computational costs of the multimodal registration upon various numbers of DDR slices to show the complex software performance. The proposed complex model has versatile usage for modeling and surgery planning of the pelvis area in fractures of iliosacral joints.

## 1. Introduction

Preoperative consideration and planning are necessary before applying any osteosynthesis. It is advisable to determine the optimal method of fracture repositioning, to determine the type of osteosynthesis and also to select the optimal osteosynthetic material for the specific case of injury. Nowadays, there are several computer software tools that allow for virtual fracture repositioning and also virtual osteosynthesis [1,2]. These software tools usually operate with CT data of the affected skeletal area. Once the CT data is loaded into the preoperative planning software, it is possible to separate the individual fracture fragments. Some software tools can separate larger fragments automatically; correction by the program user is necessary to refine the boundaries of the fragments. In most cases, point marking of the fragment margins is necessary, and the software then allows for the margins to be drawn more accurately than with automatic separation. After the separation of individual fragments, which are distinguished by color, their repositioning is possible. Repositioning can be done manually again (using the mouse), where most of the software allows for moving the fragments in different axes and planes. Some software allows for automatic repositioning using mirroring. These alternatives require a CT scan of the healthy part of the skeleton—the other side of the pelvis, the other limb. After repositioning, it is possible to insert virtual osteosynthetic material plates, screws, nails, etc. However, most of the software is tied to a specific manufacturer of osteosynthetic material, which will supply the software with the necessary shape and dimensions of the individual components. The software also allows for the optimal shaping of osteosynthetic material-especially plates. The virtual plate thus shaped can be removed from the software and sent to the manufacturer to produce an individually shaped implant. The optimal dimensions of individual plates, screws or nails can be determined in the software. The planning of the implant placement is also possible in the computer navigation software preoperatively, but the time for this task is limited as it may increase the total operating time.

In cooperation with the Technical University in Ostrava, Czech Republic and Trauma Center at the University Hospital in Ostrava, software tools were developed that would link preoperatively planned implant placement based on CT data with preoperative fluoroscopic X-ray projections. After fracture repositioning and implant planning, this proposed software would be used to fit the X-ray fluoroscopic projections to the prepared preoperative model. The proposed software tools would then allow the planned implant to be implanted into the fluoroscopic projections. During surgery, it would be possible to select the optimal skeletal entry for the insertion of guide wires or canal drilling and also to compare the direction of drilling with the optimal direction determined by the position of the planned implant. At present, these tasks are performed on the basis of the operator’s preoperative reasoning as well as his anatomical knowledge and imagination during the surgical procedure. The main contributions of this study include the following:

- Multiregional 3D segmentation model of pelvis area from CT images
- Reconstructed DDR projections with virtual iliosacral screw
- Multimodal (X-ray/CT) image registration for optimal CT slice selection according to the reference X-ray image.

The rest of the paper is organized as follows. In Section 2, we describe a recent state-of-the-art CAOS system in orthopedic surgery. Section 3 is focused on the description of the proposed system, and Section 4 aims to present the achieved results of the proposed system on two cases of iliosacral joint injury. Section 5 presents a discussion and future perspectives in this research.

## 2. Recent Work

Over the last decade, computer-assisted orthopedic surgery (CAOS) has become a part of orthopedic surgery, allowing surgeons to perform surgical operations with better accuracy and results, thus improving the patient’s well-being [3]. Finding its application first in orthopedic spinal surgery [4,5], CAOS has progressed into other procedures involving the musculoskeletal system, ranging from total hip or knee arthroplasty (THA and TKA, respectively) to bone tumor surgery [3,6,7]. CAOS navigation and planning are implemented using various methods and their combination, of which the major ones are CT-based imaging, intraoperative fluoroscopy-based imaging, and imageless navigation systems [3,7,8,9].

Sacroiliac joint dislocations and sacral fractures present a challenging treatment due to the proximity of neurovascular structures. The optimal treatment is done with percutaneous iliosacral screw (ISS) insertions. Computer-assisted navigation has the potential to not only reduce malposition rate and nerve and vessel injuries but also decrease operation time and radiation exposure [10]. As such, precise anatomical data of the patient’s pelvis and sacral bone are required. Virtual planning of pelvic and sacral bone operations on a patient-specific 3D model of the hip allows surgeons to compare different operation strategies.

CT-guided iliosacral fixation offers direct visualization of the screws, thus reducing the malposition of the screws and radiation dose. This procedure provides a precise geometry of fracture fragments and anatomical structures, allowing an accurate insertion [11,12,13,14].

A combination of 3D fluoroscopic navigation and computed tomography (CT-3D-fluoroscopy) was applied by inserting an iliosacral screw on three types of simulated posterior pelvic ring disruptions. Despite the small patient sample size, CT-3D-fluoroscopy proved to be successful in assisting iliosacral joint surgery with no or little damage to surrounding soft tissue [13,14,15].

CAOS procedures combined with 3D printing [16] of patient-specific pelvic bone present a potential guide template to treat iliosacral joint dislocation. The model is first reconstructed from CT images in an appropriate software (Mimics and similar), and then 3D printed with photosensitive resin. The guide template had a reduction in fluoroscopy time and screw insertion time with low blood loss and postoperative recovery [17].

CAOS has also undertaken a small part in virtual reality (VR) training simulations providing the ability to train on anatomically accurate 3D hip models for orthopedic surgeries, particularly aimed at medical residents. Compared to conventional training, simulations have the potential to create a risk-free environment to improve the personnel’s skills without any harm to patients. The main limitations of training simulators are feedback to the trainee, availability, and financial cost of such simulators [18,19].

A notable proposal is to use an arc screw for the internal fixation of a pelvic fracture through an internal arc fixation channel (IAFC) located inside the pelvis [20,21,22]. An automatic planning algorithm for pelvic fractures is introduced to determine an optimal channel for the arc screw. Utilizing a finite element analysis (FEA) on pelvic 3D models, a precise stress simulation determined the force applied to the pelvis in three types of postures. Based on the FEA results, the planning algorithm then locates the position, length and curvature of the arc screw for pelvic ring fracture fixation, which is patient-dependent. The current implementation uses a robot-navigated drilling system capable of drilling constant curvature in a Sawbone model of the pelvis. Due to the complexity of the pelvic structure and the surrounding soft tissue, the feasibility of IAFC is still subject to change [20,21,22].

## 3. Materials and Methods

**Figure 1:**
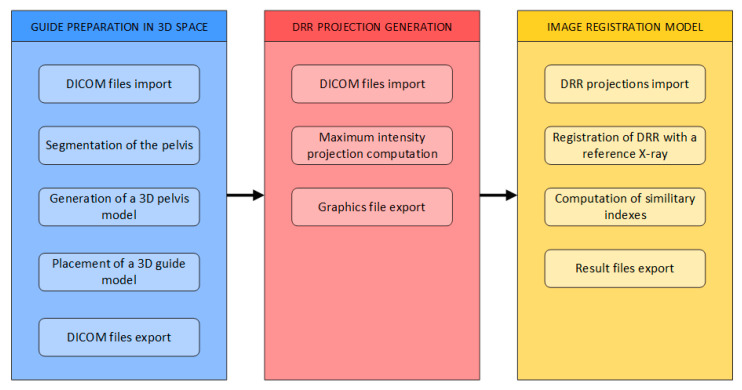
General workflow of 3D generation to image registration.

In this section, we present the realization and workflow of positioning the guide for an iliosacral screw. The proposed scheme from this study is aimed at the generation of a segmentation model in the system Mimics, reflecting individual bones in the pelvis area. Consequently, the proposed methodology generates reconstructed radiograph DDR projections from the system Mimics with Iliosacral Screw. Lastly, we propose a multimodal registration scheme, which is aimed at finding the CT slice which is the best fit for a given X-ray image for orientation in the 3D CT plane. The general workflow consists of the three mentioned phases, as shown in Figure 1:

- Generation of 3D models of the pelvis
- Generation of digitally reconstructed radiograph (DRR) projections
- Multimodal image registration of DRR projections to a reference X-ray image

### 3.1. Generation of 3D Models of the Pelvis

An image processing software Materialise Mimics generates 3D models from computed tomography (CT) image data and positions the guide 3D data optimization software. Materialise Mimics and Materialise 3-matic were used, both developed by a Belgium company Materialise NV. These software tools offer the user a wide range of tools to extract from and edit anatomical structures in medical images, generate 3D mesh models and compute various analyses and measurements. In order to create accurate 3D models and place the guide, voxels representing the pelvis are first extracted using a thresholding-based segmentation method, which compares each voxel’s Hounsfield unit (HU) value to a certain range with minimum (T_1_) and maximum (T_2_) threshold values, as shown in (1). The output is a 3D binary mask, represented as a 3D matrix, which is used with the original CT images to compute a 3D mask composed of voxels that fall within the defined range of Hounsfield units.

(1) $$I_{2}\left(x,y\right)=\left\{\begin{array}{c} 0\;\;\;\;\;\;\;\;\;\;\;\;\;\;\;\;\;\;I_{1}\left(x,y\right)>T_{2}\\ 1\;\;\;\;\;\;\;T_{1}>I_{1}\left(x,y\right)\leq T_{2}\\ 0\;\;\;\;\;\;\;\;\;\;\;\;\;\;\;\;\;I_{1}\left(x,y\right)\leq T_{1} \end{array}\right.$$

where I_1_ and I_2_ are the input and output 3D matrices consisting of CT slices. This method extracts the desired bone tissues and removes surrounding soft tissue, like muscles and skin. However, it will occasionally extract voxels that represent other anatomical structures, medical objects or scattering artifacts caused by already present metal objects whose Hounsfield unit is similar to those of bone tissues. The region-growing segmentation method is then used on each CT slice to remove these undesirable artifacts and objects, thus increasing the quality of the following 3D reconstruction. It is an iterative algorithm that grows a region of voxels depending on their seed values S_i_(x,y):

(2) $$\left|I\left(S_{i}\left(x,y\right)\right)-I\left(x,y\right)\right|\leq T$$

**Figure 2:**
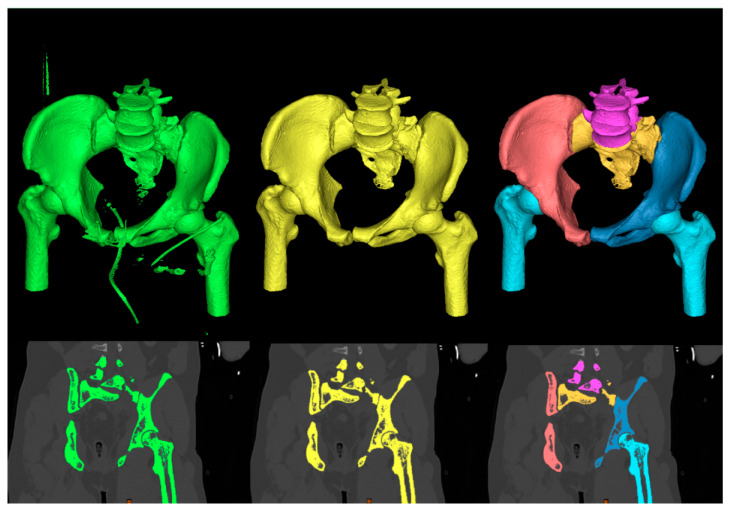
Process of segmenting and separating pelvis from CT slices into individual anatomical structures. Left to right: thresholding output (green color), region growing output (yellow color) and mask splitting output (multiple colors). The top row is a representation of masks in 3D space, and the bottom row is in 2D space.

If the result of the absolute value is greater than a threshold value T, the voxel is added to the output region. The growth is also dependent on the connectivity between voxels, where a 6-connectivity option checks neighboring faces of the selected voxel and those which are connected to it. The last operation consists of splitting the whole pelvis mask into individual anatomical structures: lumbar vertebrae, sacral bone, left and right ilium and femurs. The process of the segmentation and decomposition of the pelvis area from CT slices is presented in Figure 2.

**Figure 3:**
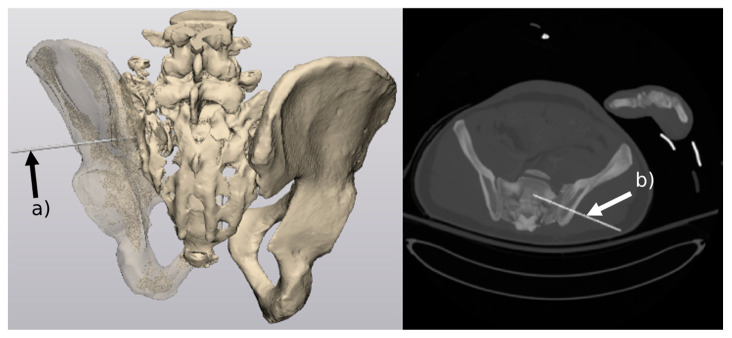
(**a**) placement of the guide in 3-matic, (**b**) maximum intensity projection (MIP) of 30 CT slices displaying the location of the guide and its insertion depth.

The purpose of splitting the mask is to give the option of hiding individual bones to accurately position the guide. From the resulting mask, we use Mimics’ marching cubes algorithm to generate a 3D mesh object of the pelvic bones. The mesh object is then exported into 3-matic (Figure 3), where the guide is positioned depending on the patient’s injured iliosacral region. Once the guide’s 3D mesh model is placed, it is exported into Mimics in an STL format, which retains information about its location and rotation in 3D world space. A new mask of the guide is then generated and fused with CT slices. Lastly, new DICOM files in the form of CT slices are generated, containing both anatomical structures and the positioned guide (Figure 3).

### 3.2. DDR Projection Generation (CT2DDR)

**Figure 4:**
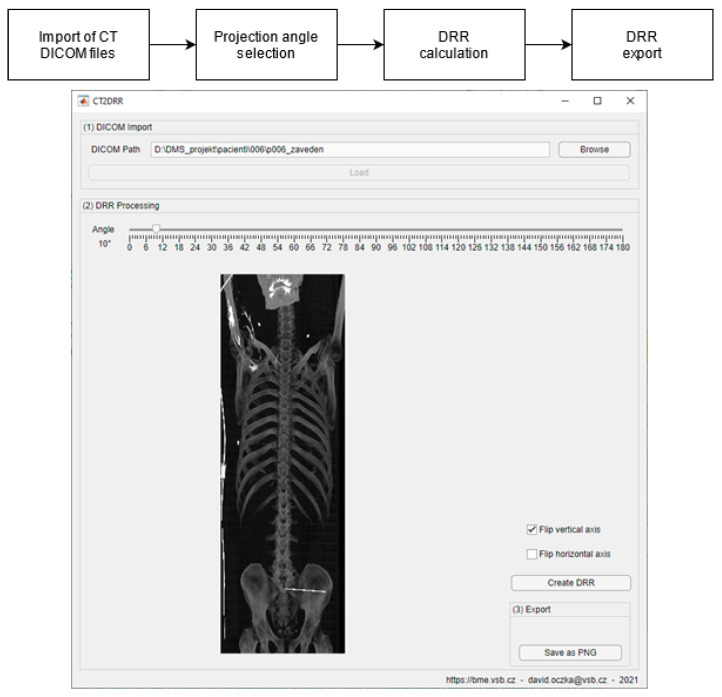
Block diagram of the process of generation DRR projections and proposed software for generating DDR projections.

Digitally reconstructed radiography (DRR) projections simulate a conventional 2D X-ray image based on CT imaging data. The projections are generated with a maximum intensity projection technique that selects the highest Hounsfield unit of each CT slice. The following section describes the process of generating DRR projections (Figure 4), consisting of two steps: image rotation and maximum intensity projection. The resulting DRR projections are then used to register a reference X-ray image. For the purpose of acquisition of DDR projections, we have developed the SW CT2DDR, which calculates individual DDR projections.

**Figure 5:**
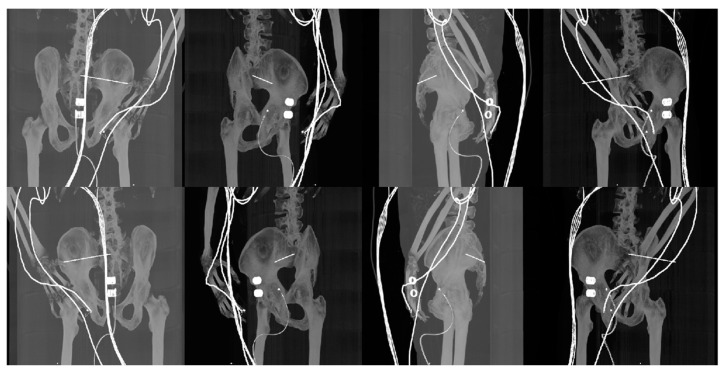
Generated series of DRR projections with rotations per 45°.

An interactive application has been created to import DICOM files, which store CT data in the form of transversal slices. First, a three-dimensional matrix consisting of ordered CT slices as layers is rotated at a specific angle θ around the z-axis. The rotation is performed using a rotation matrix R_z_:

(3) $$R_{z}\left(\vartheta \right)=\left[\begin{array}{ccc} \cos\vartheta & -\sin\vartheta & 0\\ \sin\vartheta & \cos\vartheta & 0\\ 0 & 0 & 1 \end{array}\right]$$

which is a form of image transformation that changes the location of each pixel. Second, maximum intensity projection locates the highest pixel intensity value, as described in (4), and stores it in a two-dimensional matrix, where the output matrix’s number of rows and columns correspond to the input matrix’s number of layers and columns, respectively.

(4) $$I_{x,y}=max\left(M_{x,\;y,z}\right)$$

where *M* is the input three-dimensional matrix, and *I* is the output two-dimensional matrix. The generated DRR projections are then exported as graphics files, as shown in Figure 5.

### 3.3. Image Histogram Pre-Processing

Image histogram matching is a low-level image processing transformation that aims to normalize the histogram of an input image to that of a reference image, thus changing the distribution of pixel intensities [23]. Histogram matching is used as a potential pre-processing step to increase the precision and decrease the computation time of image registration.

The algorithm is described as computing the histogram p_r_(r) of an input image and using it to its pixel values to the values in the histogram equalized image in the range k = [0, L − 1]:

(5) $$s_{k}=\left(L-1\right)\overset{k}{\underset{j=0}{\sum }}p_{r}\left(r_{j}\right)$$

where L is the maximum pixel value in the input image based on its bit depth and then computes all values of a transformation function G(z_q_) in the same range as k, so that G(z_q_) = s_k_:

(6) $$G\left(z_{q}\right)=\left(L-1\right)\overset{q}{\underset{i=0}{\sum }}p_{z}\left(z_{i}\right)$$

and obtain values z_q_ from the inverse transformation of G:

(7) $$z_{q}=G^{-1}\left(s_{k}\right)$$

The result is a histogram-matched image mapped from equalized pixel values s_k_ to the corresponding values z_q_.

### 3.4. Image Registration Model

Image registration is one of many tasks of medical image analysis, which deals with collecting, processing, and evaluating medical images acquired from imaging techniques, most prominently magnetic resonance imaging (MRI), computed tomography (CT), positron emission tomography (PET), and single photon emission computed tomography (SPECT), but also conventional radiography (CR) and medical ultrasonography (US). It is a process of transforming images or 3D volumes from the same (monomodal) or multiple modalities (multimodal) into a single coordinate space so that the data can be accurately compared and studied. Its task is to reduce inevitable misalignment, which manifests in pre, intra and postoperative image acquisition. Registration is realized by mapping the source images to target images, also called sensed images and reference images, respectively. Monomodal registration deals with images taken from the same modality by the same scanner: CT-CT or MRI-MRI. Multimodal registration, on the hand, deals with images taken from different modalities and different scanners: CR-CT, CT-MRI, or CT-PET [24,25,26].

In this paper, we introduce the use of multimodal image registration to preoperatively plan screw insertions in the iliosacral region. The first section describes the registration algorithm used to register source CT image data to target CR images. The second section describes similarity metrics used to assess the precision of registration.

#### 3.4.1. Multimodal image registration algorithm

Image registration algorithms [26] can be defined by three components. First is a cost function that describes the dissimilarity between two images and is formed by various regularization terms, such as fluid, diffusion and elastic. Second is a space of geometric transformations, which allows the images to deform. These are rigid for translation, rotation and scaling and affine (non-rigid) for additional warping. The third component is a strategy for minimizing the cost function. This paper focuses on the implementation of Thirion’s [27,28,29,30] Demons algorithm of image matching as a diffusion process. It is an optical flow-based affine registration method that computes the demon forces according to the local characteristics of the images. Gaussian smoothing filter with a given σ is then used as a regularization term for each iteration until convergence. The process is based on the following optical flow equation, which describes the displacement $\vec{v}$:

(8) $$\vec{v}=\frac{\left(m-s\right)\cdot {\vec{\nabla }}_{s}}{{\left(\vec{\nabla_{s}}\right)}^{2}+{\left(m-s\right)}^{2}}$$

where m and s are intensity functions of the source image M and the target image S, respectively, at a certain point and ${\vec{\nabla }}_{s}$ is a gradient of the source image S.

**Figure 6:**
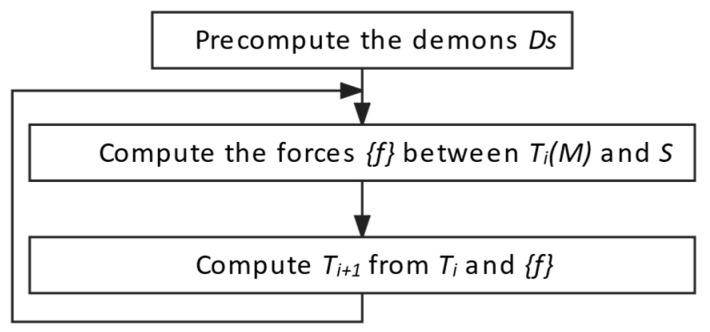
Block diagram of the multimodal registration algorithm for matching X-ray and CT images.

Considering a source image M and a target image S, the algorithm aims to find a final transform T that belongs to a set of allowed deformations T between the space M of the source image M and the space S of the target image S. In each iteration, the deformed T_i_(M) of the source M becomes T_i+1_(M), constrained internal forces fint and external forces fext, created by the interactions between T_i_(M) and S. This process is described in a block diagram in Figure 6.

**Figure 7:**
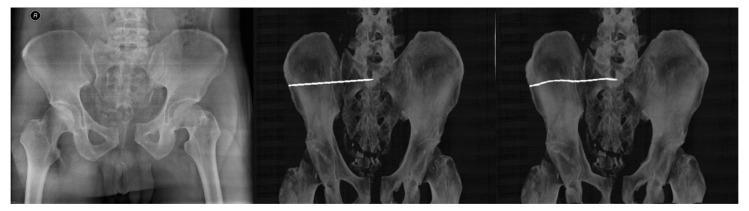
Result of Demons algorithm for multimodal image registration. (**Left**, **Middle**, **Right**): Target (reference) X-ray image, moving (input) DRR projection image, the output of image registration.

The first step consists of precomputation of the set of demon forces *Ds*, which are extracted from the target image *S*, where one pixel (voxel for 3D images) corresponds to one demon force. The second step is an iterative estimation of the deformation of the source image *T*, from the source space *M* to the target space *S*. The demon force can be described by its spatial position *P* or intensity at that location *s(P)* or a direction from the inside to the outside based on the gradient. Figure 7 presents the example of using the multimodal registration between the target (X-ray) image and moving (DDR projections).

#### 3.4.2. Statistical Metrics for Registration Evaluation

Structural similarity index [31] or SSIM index is a metric for the objective evaluation of two images containing the same overall structures, which are defined by their contrast, shape and luminance. This metric is defined as:

(9) $$SSIM\left(x,y\right)=\frac{\left(2\mu_{x}\mu_{y}+C_{1}\right)\left(2\sigma_{xy}+C_{2}\right)}{\left(\mu_{x}^{2}+\mu_{y}^{2}+C_{1}\right)\left(\sigma_{x}^{2}\sigma_{y}^{2}C_{2}\right)}$$

where *μ* is the weighted average of images *x* and *y*, and *σ* is the covariance of *x* and *y*. Parameter *C_i_ = (K_i_*, *L)^2^*, where *L* is the dynamic range of pixel values (2^n-bits/pixel^ – 1) and *K***_1_** ≪ 1 and *K*_2_ ≪ 1 are scalar constants for *C*_1_ and *C*_2_. The resulting index value lies within the range of [0, 1], where SSIM values at 0 indicate the lowest similarity and values at 1 indicate the highest similarity of input images *x* and *y*.

A correlation coefficient is a metric that computes the linear correlation between two images, *x* and *y*, defined as the ratio of the sum of multiplied differences and squared root of multiplied sums of squared differences:

(10) $$CORR\left(x,y\right)=\frac{\sum \left(x_{i}-\bar{x}\right)\cdot \left(y_{i}-\bar{y}\right)}{\sqrt{\sum {\left(x_{i}-\bar{x}\right)}^{2}\cdot \sum y_{i}-{\bar{y}}^{2}}}$$

where *x_i_* and *y_i_* are pixel intensity values, $\bar{x}$ and $\bar{y}$ are the arithmetic mean of each image. The resulting value lies within the range of −1 and +1, where CORR below 0 indicates a lower linear correlation and above 0 indicates a higher linear correlation between images *x* and *y*.

The resulting registered DRR projections are evaluated in terms of similarity to their respective reference X-ray image. The higher the evaluation indexes, the better the registration algorithm deformed DRR projections to their reference X-ray image.

## 4. Results

This section is focused on the results of image registration with the use of the Demons algorithm to match input DRR projection images to reference X-ray images. Computed tomography and X-ray datasets of two patients have been used in this pilot study. The patient’s records were used in this study under the approval of the Ethics Committee of the University Hospital in Ostrava, Czech Republic, with reference number: 1030/2022. Table 1 and Table 2 describe the technical parameters of each image dataset per patient.

**Figure 8:**
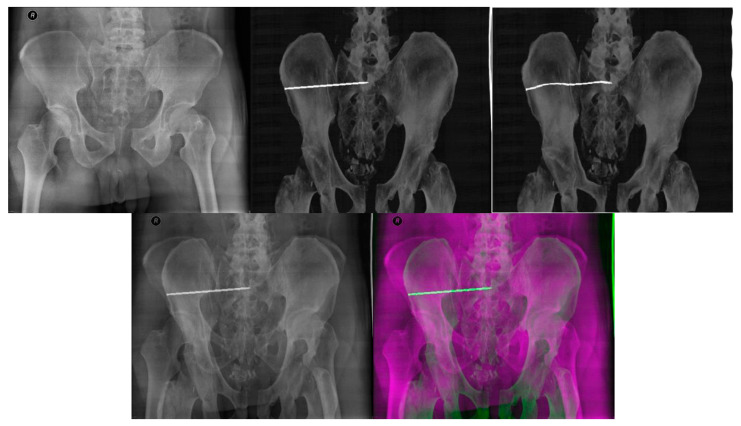
The output of image registration for Patient 1. **Left** to **Right**: Reference X-ray image, input DRR projection, output DRR projection, alpha blending composite image and RGB composite image.

**Figure 9:**
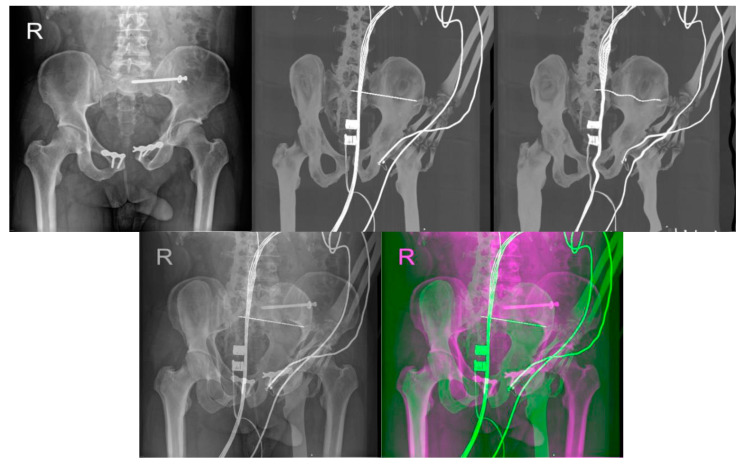
The output of image registration for Patient 2. **Left** to **Right**: Reference X-ray image, input DRR projection, output DRR projection, alpha blending composite image and RGB composite image.

The registration process follows the steps described in Figure 1. A corresponding range of DRR projections is chosen, depending on the position and rotation of the patient’s pelvis on the X-ray image. This range of projections considers any deviations in the patient’s position and rotation which are likely to occur during X-ray and CT scans. A smaller range of projections, 10° for example, is preferred to reduce the computation time of registration. The output registration images are then evaluated using similarity metrics described in statistical evaluation metrics to obtain the projection with the highest similarity to its reference image, as seen in Figure 8 and Figure 9, where the registered and its input image have the highest similarity.

**Figure 10:**
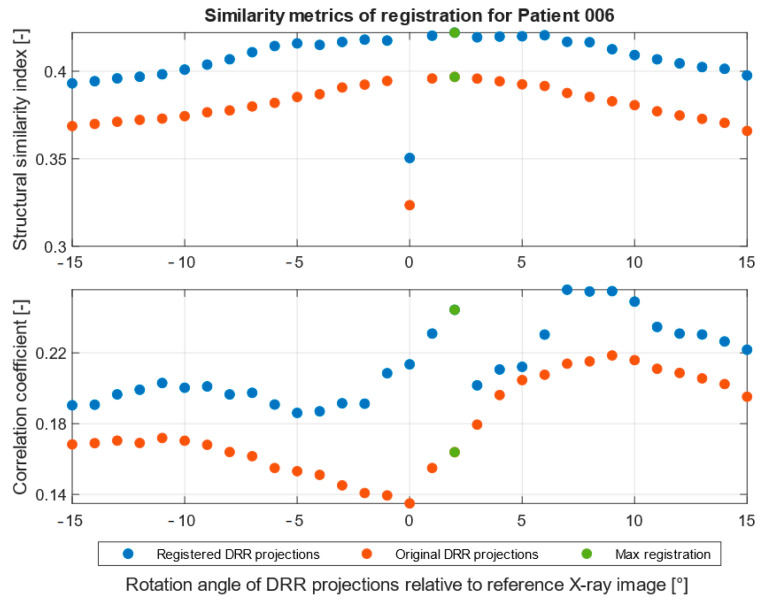
Similarity metrics of image registration of 30° angle range for Patient 1. DRR projection with the highest similarity is marked as green.

**Figure 11:**
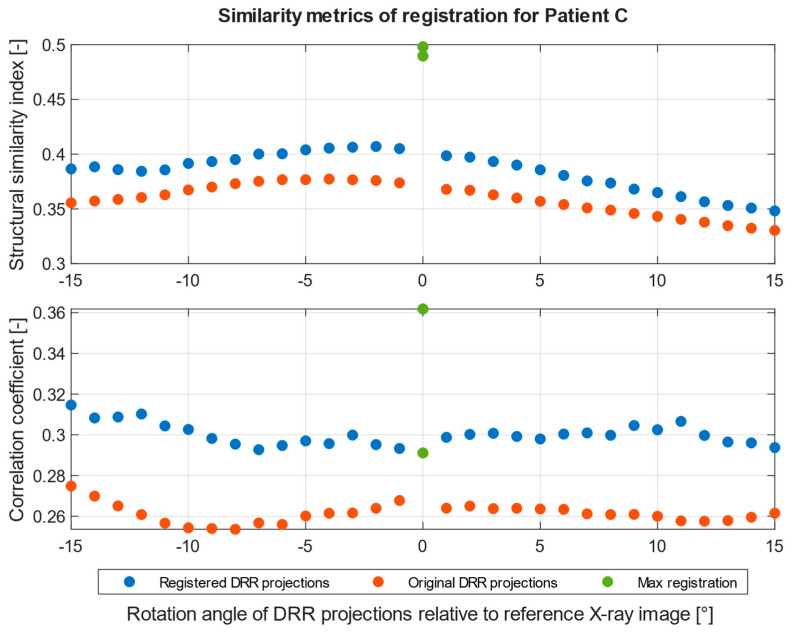
Similarity metrics of image registration of 30° angel range for Patient C. DRR projection with the highest similarity marked as green.

Figure 10 and Figure 11 show the summary of similarity metrics per a normalized rotation angle of a registered and an original DRR projection for two patients, where angle 0° signifies the base DRR projection, which corresponds to the general rotation of the patient’s pelvis on the reference X-ray image, 345° is the lower limit, and 15° is the upper limit of a 30° angle range. After normalization, the range spans from −15° to 15°, with 0° being the base DRR projection. A full summary of the highest similarity results and their projection angle per patient can be found in Table 3. Due to the difference in computing the structural similarity index and correlation coefficient, during which the correlation coefficient computes only the mutual relationship of pixel values of two images, the resulting registered DRR projection with the highest similarity to the reference image may differ. This can be observed in Figure 10 of the results for Patient 1, where the best-matched projection angle based on structural similarity is 2°, unlike the correlation coefficient, which is 8°. The structural similarity index and correlation coefficient in Figure 11 of patient 2 results correspond. The difference in the highest results is mainly dependent on pixel intensity distributions of DRR projection images and X-ray images and differs across the tested patient datasets. Therefore, we indicate the best-registered projection angle for both metrics based on the highest value of the two metrics and the nearest projection angle to the base projection angle, which must correspond to the rotation on the patient’s X-ray image.

### Computation Time

Overall computation time is significantly dependent on the range of DRR projections to be registered, the size and pixel intensity distribution of input and reference images and the computer hardware used. We used two computer systems to compare this. The first computer, designated as PC 1, consists of the following components: 6th generation quad-core Intel Core i5-6400 processor with a frequency of 2.7 GHz, 16 GB of available memory, Nvidia GTX 1060 6 GB graphics card and Windows 10 operating system. The second system, PC 2, consists of a 10th generation 8-core Intel Core i5-10300H processor with a frequency of 2.5 GHz, 16 GB of available memory, Intel UHD Integrated and Nvidia RTX 2060 6 GB graphics cards and Windows 10 operating system.

On PC 1, the registration time lies in the range of 7.65 and 9.22 s, with a mean of 7.74 s per DRR projection for Patient 1. On PC 2, the average time had been reduced to 6.36 s per DRR projection in an overall range of 5.64 and 7.39 s. A summary of computation times for both computer systems per patient is shown in Table 4.

**Figure 12:**
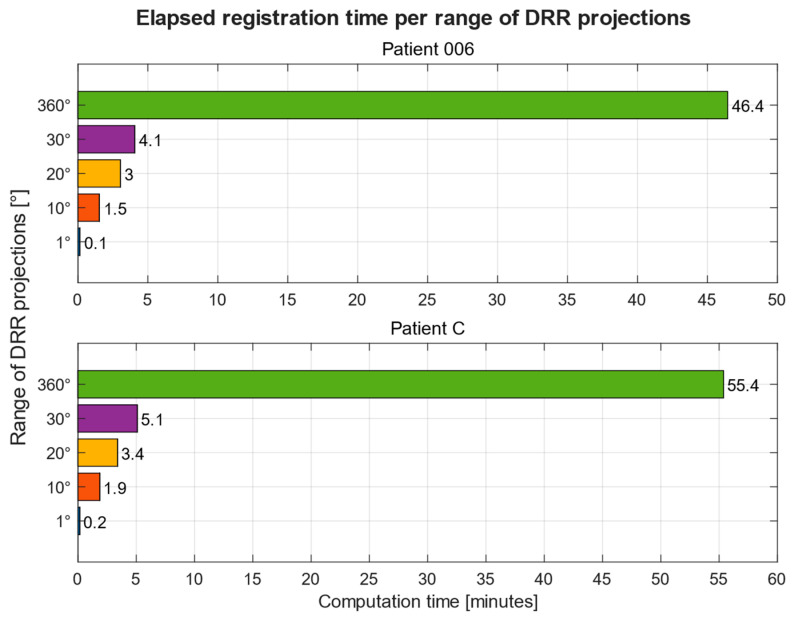
Computation times in minutes of image registration per range of rotation angles of DRR projections for two patient datasets. Acquired on PC 1.

Figure 12 shows the computation times of registration of 5 ranges of DRR projections, 1°, 10°, 20°, 30°, and 360°, for two patients on PC 1. A full summary of computation times per these ranges across all patients and both computer systems can be found in Table 5.

As described in the pre-processing section, image histogram matching has been used to potentially improve the overall registration in terms of precision and computation time. This was tested on two datasets on PC 1. The summary of the results can be seen in Table 5.

**Figure 13:**
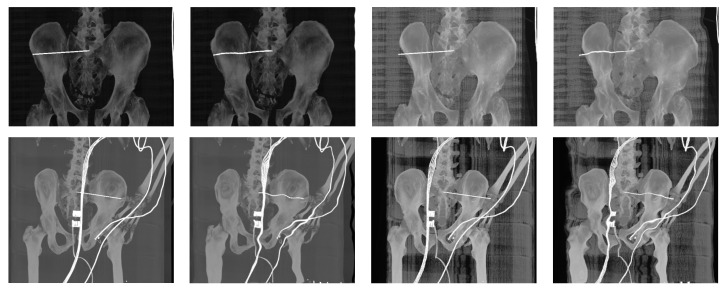
Best registered DRR projections with and without histogram matching. (**Top Row**) is Patient 1, (**Bottom Row**) is Patient 2.

Despite the input DRR projections having a more uniform pixel value distribution to its reference X-ray image, we found minor improvements of similarities of Patient 1 at the cost of a 0.3 s increase in mean computation time. For Patient 2, however, there was a decrease in similarities with lower mean computation time, 0.18 s difference. On both occasions, the best-registered projection resulted in the angle range limits, at 15° for Patient 2 and at 345° (normalized—15°) for Patient 2, which greatly differ from those without histogram matching. Figure 13 shows the difference in the pixel intensity distribution of both datasets. Reference X-ray images are the same as in Figure 8 and Figure 9, respectively.

## 5. Discussion and Conclusions

In this paper, we describe a novel technical solution for the simulation of planning of iliosacral screw placement for iliosacral joint injuries. This system is able to virtually simulate placing an iliosacral screw into preoperative CT 3D scans in the form of DDR projections and classify respective CT scans with the screw according to the intraoperative X-ray images. This complex procedure enables surgeons to investigate the operation area with a virtual screw anytime during the surgery and guide them to optimal lead the iliosacral screw to avoid other surrounding tissues.

The proposed complex software solution enables the modeling of the pelvis area as a multiregional segmentation procedure (Figure 2) using the Region growing method. This procedure consequently allows for the virtual placing of the iliosacral screw (Figure 3), which is consequently used for the generation of DDR projections. These projections represent a 3D model of the investigated pelvis area for spatial manipulation with the screw in 3D space instead of 2D X-ray images. To find the most suitable 3D slice which best corresponds with the reference X-ray image, we incorporated a multimodal registration procedure, which is aimed at finding the 3D DDR slice which best corresponds with a reference X-ray image (Figure 7).

This is a versatile procedure, which enables surgeons to make a 3D model of the pelvis area and virtually place an iliosacral screw in the 3D space, with the consequent generation of DDR projections, for which we created in SW MATLAB the application CT2DDR (Figure 4). This procedure can be versatile and applied for any X-ray image with using of the proposed multimodal (X-ray/CT) registration, which is aimed at the selection of the 3D slice which best corresponds with the reference X-ray image. This complex procedure provides an effective transformation of 2D X-ray views, which are taken during surgery, into 3D space for better spatial orientation and planning of surgery within the preoperative phase. We built the proposed registration system based on the SSIM and correlation index, where these parameters are capable of selecting the best 3D slices, which are the most similar to a reference X-ray image (Figure 10 and Figure 11).

**Figure 14:**
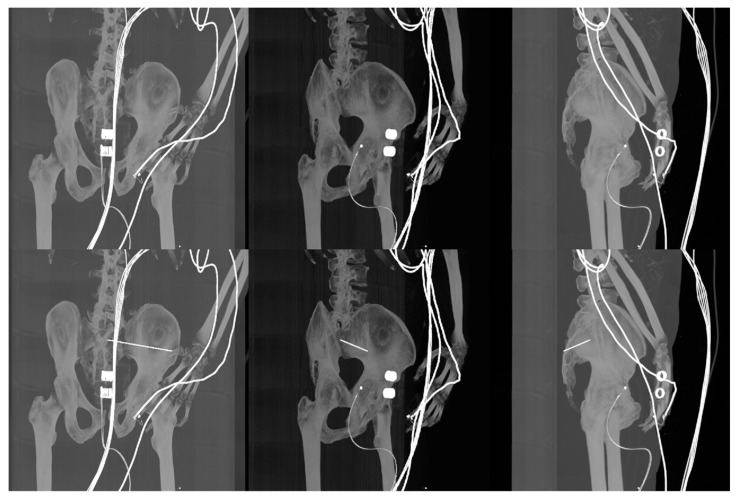
Comparison of three various CT slices (**Top Row**) of patient 2 with the same DDR projections with virtual iliosacral screw (**Bottom Row**).

In our study, we compared the native intraoperative X-ray images with selected reconstructed DDR slices with the iliosacral screw, where we pointed out the differences, which show the multimodal registration performance (Figure 8 and Figure 9). On the other hand, it is clinically important to have a comparison between the original CT slices and reconstructed DDR projections with the iliosacral screw to investigate whether the screw is safely led out of the surrounding critical tissues, as we report in Figure 14.

An important aspect of the registration procedure is computing time. Here, we publish the comparative analysis of computing costs for the various number of 3D projections (Figure 12), which shows that the number of projections is crucial for speeding up the whole registration process. This is one of the limitations of a multimodal registration procedure. For this reason, it is beneficial to select a narrower range of 3D slices for the registration to save computational costs.

The presented study represents a pilot study, which brings complex proposed software tools for planning iliosacral screw placement for iliosacral joint injuries. This pilot study serves for initial testing on two clinical cases, as we report in this paper, to justify the initial performance and usability of this system in the clinical practice of traumatology. Despite the limitation of the number of patients in this study, we achieved favorable results, which predetermine the potential of using this system in clinical conditions.

At the present time, the proposed software system is being used for the iliosacral joint fracture reposition surgery at University Hospital in Ostrava. The system brings significant benefits as the investigation of iliosacral screw in 3D space and automatic settings of a view with the screw according to the reference X-ray image, which is standardly done by C-arm during the surgery. On the other hand, we are aware of the limitations, which will be the focus of the future improvement of this system. Despite having this planning system with augmented reality in the form of an iliosacral screw, it will be beneficial to incorporate an attention-based system, which will predict the potential damage of surrounding tissues by leading the screw. Here, we plan to implement segmentation of surrounding tissues and check whether the screw is intersected with any of these segmented tissues. As a part of this procedure, we plan to implement the screw trajectory optimization technique, which should be able to predict the best way to lead the screw with minimal damage to surrounding tissues.

## Figures and Tables

**Table 1:** Technical and metadata information of X-ray image datasets per patient.

	Imaging Device	Modality	Bit-Depth	Image Resolution [Pixels]	Pixel Spacing [mm]	View Position	Body Part Examined
Patient 1	Kodak Elite CR	CR	16	2048 × 2500	0.17/0.17	AP	Pelvis
Patient 2	Samsung GC85	DX	16	2994 × 2990	0.13/0.13	AP	Pelvis

**Table 2:** Technical and metadata information of computed tomography image datasets per patient.

	Imaging Device	Modality	Bit-Depth	Image Resolution [Pixels]	Pixel Spacing [mm]
Patient 1	Siemens Definition AS	CT	16	512 × 512	0.81/0.81
Patient 2	Siemens Somatom Force	CT	16	512 × 512	0.94/0.94
	Convolution kernel	Pitch factor [mm]	Number of slices	Slice thickness [mm]	Body part examined
	B20f	1.05	1561	0.6	Abdomen
	Br40d/2	1.4	779	0.75	Abdomen

**Table 3:** Summary of highest similarity results and their DRR projection per patient.

	Patient 1	Patient 2
SSIM [-]	0.42	0.50
CORR [-]	0.26	0.36
DRR projection	2°	0°

**Table 4:** Summary of computation times in minutes of image registration per range of rotation angles of DRR projections across all patient datasets.

	Computation Times on PC 1		Computation Times on PC 2	
DRR Projections	Patient 1	Patient 2	Patient 1	Patient 2
1°	0.13	0.15	0.11	0.13
10°	1.53	1.87	1.32	1.36
20°	3.02	3.40	2.23	2.64
30°	3.93	4.64	3.26	3.89
360°	46.45	55.39	38.24	45.15

**Table 5:** Summary of similarity metrics and mean computation times of two datasets in an angle range of 30°.

	Without Histogram Matching				With Histogram Matching			
	SSIM [-]	CORR [-]	Mean Time [Seconds]	DRR Projection	SSIM [-]	CORR [-]	Mean Time [Seconds]	DRR Projection
Patient 1	0.42	0.26	7.87	2°	0.51	0.42	8.17	15°
Patient 2	0.50	0.36	9.86	0°	0.40	0.42	9.68	345°

## Data Availability

The CT and X-ray images and software CT2DDR from this research will be available upon request from the corresponding author.
